# Top-Down and Intact Protein Mass Spectrometry Data Visualization for Proteoform Analysis Using VisioProt-MS

**DOI:** 10.1177/1177932219868223

**Published:** 2019-08-16

**Authors:** Jean Lesne, Marie-Pierre Bousquet, Julien Marcoux, Marie Locard-Paulet

**Affiliations:** 1 Institut de Pharmacologie et de Biologie Structurale, Université de Toulouse, CNRS, UPS, Toulouse, France; 2 Disease Systems Biology Program, Novo Nordisk Foundation Center for Protein Research, University of Copenhagen, Copenhagen, Denmark

**Keywords:** Mass spectrometry, proteasome, top-down, LC-MS, intact protein MS

## Abstract

The rise of intact protein analysis by mass spectrometry (MS) was accompanied by an increasing need for flexible tools allowing data visualization and analysis. These include inspection of the deconvoluted molecular weights of the proteoforms eluted alongside liquid chromatography (LC) through their representation in three-dimensional (3D) liquid chromatography coupled to mass spectrometry (LC-MS) maps (plots of deconvoluted molecular weights, retention times, and intensity of the MS signal). With this aim, we developed a free and open-source web application named VisioProt-MS (https://masstools.ipbs.fr/mstools/visioprot-ms/). VisioProt-MS is highly compatible with many algorithms and software developed by the community to integrate and deconvolute top-down and intact protein MS data. Its dynamic and user-friendly features greatly facilitate analysis through several graphical representations dedicated to MS and tandem mass spectrometry (MS/MS) analysis of proteoforms in complex samples. Here, we will illustrate the importance of LC-MS map visualization to optimize top-down acquisition/search parameters and analyze intact protein MS data. We will go through the main features of VisioProt-MS using the human proteasomal 20S core particle as a user-case.

**Comment on:** Locard-Paulet M, Parra J, Albigot R, et al. VisioProt-MS: interactive 2D maps from intact protein mass spectrometry. *Bioinformatics*. 2019;35(4):679-681. doi:10.1093/bioinformatics/bty680. PubMed PMID:30084957. PubMed Central PMCID:PMC6378940. https://www.ncbi.nlm.nih.gov/pmc/articles/PMC6378940/.

## Introduction

Top-down proteomics consists in the analysis of intact proteins using liquid chromatography coupled to mass spectrometry (LC-MS), followed by their identification by tandem mass spectrometry (MS/MS). This informs on the protein composition of the analyzed sample, and their potential combinations of post-translational modifications, splicing events, and/or mutations. Intact protein mass spectrometry (MS) recently increased in throughput^1^ and became quantitative,^2,3^ thereby allowing the in-depth characterization of proteoforms^4^ in complex samples.^5^ Such development was associated with the establishment of a specific lexicon,^6,7^ dedicated databases (repository.topdownproteomics.org), and, needless to say, a panel of bioinformatics tools.^8-11^

Typically, top-down and intact protein MS analysis relies on the measurement of the deconvoluted molecular weights (MWs) of proteoforms after separation by liquid chromatography (LC). This can be facilitated by the graphical representation of LC-MS three-dimensional (3D) maps, where the *x*-axis represents retention time (RT), the *y*-axis represents deconvoluted MW, and the color represents the intensity of the MS signal. In addition, directly comparing these maps reveals differences in proteoform footprints between samples and/or experimental conditions.^1,8,12–16^ Recently, we developed a free standalone tool to facilitate this analytical step: VisioProt-MS.^17^ Here, we will illustrate its use through the analysis of the 20S core particle of the human proteasome.

The proteasome is a ubiquitous macromolecular barrel-shaped complex of around 700 kDa that is responsible for protein degradation in eukaryotic cells.^18^ It is vital to maintain protein homeostasis and the pool of free amino acids available for protein synthesis. It also contributes to the immune response through production of antigenic peptides. Its catalytic activity resides in the 20S core particle that is composed of 4 rings of 7 subunits each: two β-rings (β1-7) surrounded by two α-rings (α1-7). The names and theoretical MWs of these subunits are presented in Table 1. β1, β2, and β5 are the only subunits that are catalytically active in the standard 20S (std20S), and they can be replaced by β1i, β2i, and β5i to form the immunoproteasome (i20S) in the context of immune response. This leads to 2 different functional complexes that have distinct catalytic activities.^19^ The proteasome is highly studied in academia and industry in the context of drug development. The 20S core particle can be immunopurified using the anti-α2 antibody MCP21,^20,21^ and as it is constituted of subunits of ~20 to ~30 kDa, it is specifically suited for top-down and intact protein MS analysis.^8^ We present here the comparative top-down analysis of in-house-produced std20S and commercial samples of purified std20S and i20S using VisioProt-MS.

**Table 1.** Theoretical molecular weights of the subunits composing the 20S core particle of the proteasome.

Subunit name	Gene name	UniProt accession	Sequence	Theoretical MW (Da)	-Met	Mature protein	-Met + 1ac	+1ac	-Met + 1ac + 1phos
α1	*PSMA6*	P60900	2-246	27 399.45	27 268.26		**27 310.27** ^a,b,c^	27 441.46	
α2	*PSMA2*	P25787	2-234	25 898.59	25 767.40		**25 809.41** ^a,b,c^	25 940.60	
α3	*PSMA4*	P25789	2-261	29 483.81	29 352.62		**29 394.63** ^a,b,c^	29 525.82	
α4	*PSMA7*	O14818	2-248	27 886.85	27 755.66		**27 797.67** ^b^	27 928.86	
α5	*PSMA5*	P28066	1-241	26 411.03				**26 453.04** ^a,b,c^	
α6	*PSMA1*	P25786	1-263	29 555.59				**29 597.60** ^a,b,c^	
α7	*PSMA3*	P25788	2-255	28 433.23	28 302.04		**28 344.05** ^b,c^	28 475.24	**28 424.02** ^a,b,c^
**β1**	*PSMB6*	P28072	35-239	25 357.72		**21 903.89** ^a,b^		21 945.90	
**β1i**	*PSMB9*	P28065	21-219	23 264.30		**21 276.05** ^c^		21 318.06	
**β2**	*PSMB7*	Q99436	44-277	29 965.42		**25 294.99** ^a,b,c^		30 007.43	
**β2i**	*PSMB10*	P40306	40-273	28 936.30		**24 648.28** ^c^		28 978.31	
β3	*PSMB3*	P49720	2-205	22 948.88	22 817.69		**22 859.70** ^a,b,c^	22 990.89	
β4	*PSMB2*	P49721	1-201	22 836.28	22 705.09		22 747.10	**22 878.29** ^a,b,c^	
**β5**	*PSMB5*	P28074	60-263	28 480.28		**22 458.37** ^a,b,c^		28 522.29	
**β5i**	*PSMB8*	P28062	73-276	30 354.26		**22 659.61** ^c^		30 396.27	
β6	*PSMB1*	P20618	29-241	26 489.37		**23 548.94** ^a,b,c^		26 531.38	
β7	*PSMB4*	P28070	46-264	29 204.24		**24 391.78** ^a,b,c^		29 246.25	

Abbreviation: MW, molecular weight.

“-Met”: loss of *N*-terminal methionine; “Mature protein”: loss of *N*-terminal propeptide; “ac”: acetylation; “phos”: phosphorylation.

**Figure 1.**
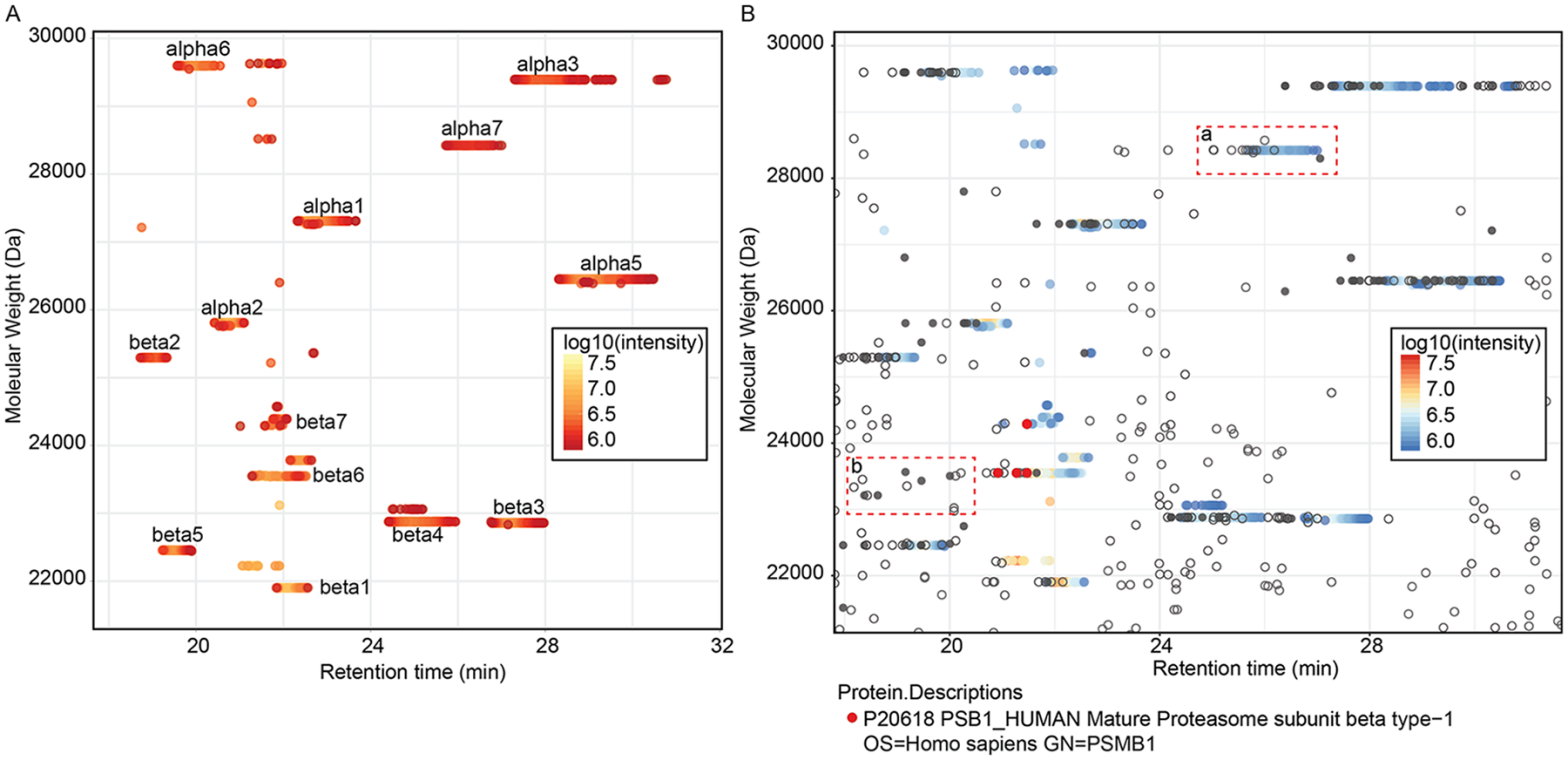
Top-down analysis of immunopurified std20S: (A) 3D proteoform footprint of the LC-MS trace of 20S subunits generated with VisioProt-MS. The names of the std20S subunits are indicated next to the corresponding signal. (B) The same sample analyzed with MS/MS fragmentation for identification and visualized with the MS/MS mode of VisioProt-MS. The MS trace is color-coded with the «Red/yellow/blue» scale, and the MS/MS identified as β6 are indicated in red. Gray empty and filled circles are MS/MS that were not identified or proteoform-spectrum matches, respectively. The 2 rectangular selections (in red dashed lines) highlight (a) a non-identified proteoform (corresponding to the phosphorylated α7—based on its MW), and (b) potential false identifications (filled circles that do not overlap with a clear deconvoluted MS trace). Both figures were drawn with a VisioProt-MS «Threshold» parameter of 80%. LC-MS indicates liquid chromatography coupled to mass spectrometry; MS, mass spectrometry; MW, molecular weight.

**Figure 2.**
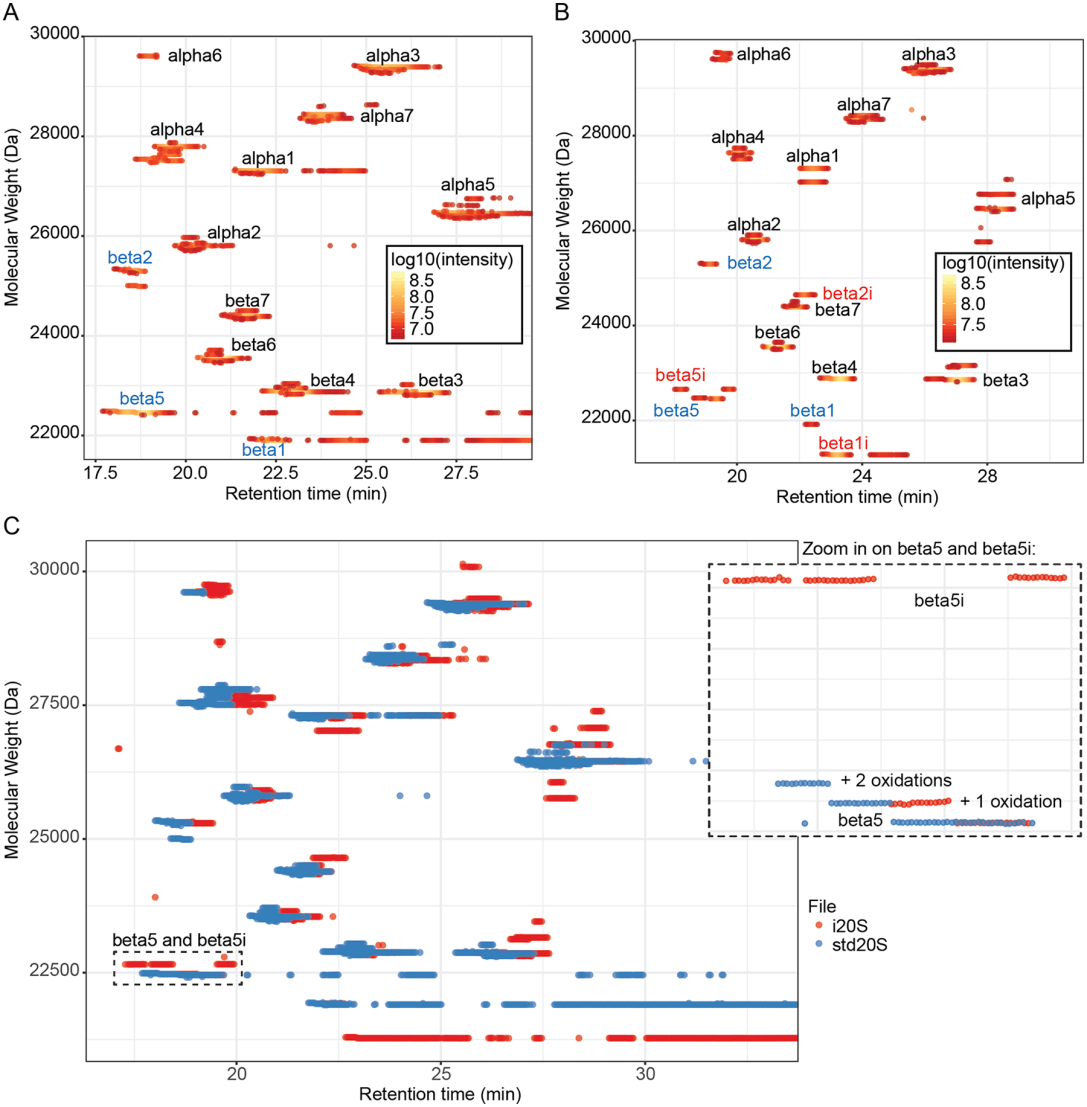
Comparison of commercial std20S and i20S using intact protein MS: (A, B) MS trace of the subunits of the std20S (A, threshold: 35%) and i20s (B, threshold: 16%) visualized with VisioProt-MS in MS mode. The names of the identified subunits are indicated next to the corresponding signal. The subunits that are specific to the standard or immunoproteasome are indicated in blue and red, respectively. (C) Overlay of the same samples for comparison of their proteoform composition (threshold: 35%). The MS trace is color-coded in function of the sample. A magnification of the area indicated with the dashed rectangular selection (“beta5 and beta5i”) is presented on the right. The names of the identified subunits are indicated next to the corresponding signal. The 3 beta5 traces correspond to the mature protein with 0, 1, or 2 oxidations. MS indicates mass spectrometry.

β1/β2/β5 and β1i/β2i/β5i are specific subunits of the std20 and i20S, respectively. The most abundant proteoforms identified in Figures 1 and 2A and B are indicated in bold with the superscripts a, b, and c, respectively.

## Material and Methods

### Reagents

All reagents were provided by Sigma-Aldrich unless otherwise specified. Commercial standard and immuno-20S were purchased from Enzo Life Science.

### In-house purification of endogenous 20S

Endogenous 20S was purified from Hek293-EBNA cells grown to 80% confluency in Iscove’s Modified Dulbecco’s Medium (IMDM; Thermo Fisher) supplemented with 10% fetal bovine serum (FBS), 116 mg/mL L-Arginine, and 36 mg/mL L-asparagine (Acros Organics) at 37°C and 5% CO_2_. Cells were then washed twice with phosphate-buffered saline (PBS) and stored at ‒80°C. On the day of experiment, 50 × 10^6^ cells were lysed with 2 mL of lysis buffer (10 mM HEPES with pH 7.9, 10 mM KCl, 5 mM MgCl_2_, 10% glycerol, 10 mM adenosine triphosphate [ATP], 1% NP40, protease Complete and phosphatase PhosSTOP inhibitors from Roche), incubated for 15 minutes at 4°C, and sonicated (Bioruptor Plus; Diagenode). Non-soluble debris were removed by centrifugation (4000*g* for 15 minutes at 4°C) and the protein concentration in the supernatant was determined by detergent-compatible (DC) assay (Bio-Rad). Aliquots were kept at ‒80°C until analysis. Immunopurification of the endogenous 20S core proteasome was performed as described in Fabre et al.^21^

### Intact protein and top-down MS analysis

Nano-LC-MS and MS/MS analyses of commercial or immunopurified 20S were performed on a nanoRS UHPLC system (Dionex) coupled to an LTQ-Orbitrap Fusion Tribrid mass spectrometer (Thermo Fisher Scientific). A total of 5 μL of sample at 0.3 µM was loaded onto a reverse-phase C4-precolumn (300 μm i.d. × 5 mm; Thermo Fisher Scientific) at 20 μL/min in 2% acetonitrile (ACN) and 0.2% formic acid (FA). After 5 minutes of desalting, the precolumn was switched online with an analytical C4 nanocolumn (75 μm i.d. × 15 cm; in-house packed with C4 Reprosil) equilibrated in 95% solvent A (5% ACN, 0.2% FA) and 5% solvent B (0.2% FA in ACN). Proteins were eluted using a binary gradient ranging from 5% to 40% (5 minutes) and then 40% to 99% (33 minutes) of solvent B at a flow rate of 300 nL/min. For the commercial 20S samples, the Fusion Tribrid (Thermo Fisher Scientific) was operated in single MS acquisition mode with the Xcalibur software (Thermo Fisher Scientific). The spray voltage was set to 1900 V, the ion transfer tube temperature to 300°C, the RF lens to 60%, and the in-source dissociation to 50 V. The MS scans were acquired in the 700 to 2000 m/z range with the resolution set to 60 000 and using 10 µscans for averaging. For the intact protein and top-down MS analysis of the immunopurified std20s, the spray voltage was set to 1350 V, the ion transfer tube temperature to 270°C, the RF lens to 60%, and the in-source dissociation to 50 V. The MS and MS/MS scans were acquired in the 400 to 2000 m/z range with a resolution of 120 000 and using 3 µscans for averaging. Ions of interest were selected according to an inclusion list of 14 precursor masses (corresponding to the 20S subunits) that were analysed by MS/MS with the option “DDA if parent mass list not found” unchecked. The isolation window was set to 5 Th with electron-transfer/higher-energy collision dissociation (EThcD) fragmentation (electron-transfer dissociation [ETD]: 20 ms and higher-energy collision dissociation [HCD]: 25%).

### Data analysis and visualization

For MS traces, raw files were automatically deconvoluted with the rolling window deconvolution software RoWinPro^8^ and the proteoform footprints were visualized with VisioProt-MS v2.0.^17^ Top-down raw data were analyzed with Proteome Discoverer v2.2 (Thermo Scientific) using the ProSight PD Top-Down Low/High node. Briefly, intact protein spectra were deconvoluted with ReSpect (precursor mass between 20 000 and 30 000 Da and 100 ppm mass tolerance, charge state range between 15 and 35). Tandem mass spectrometry spectra were deconvoluted with Xtract (S/N threshold = 3, m/z range between 200 and 2000 Da, 60 000 resolution) and searched against a custom database including all human 20S subunits (generated in ProSight PC v4.0, Thermo Scientific). The search was performed in absolute mass mode with a fragment mass tolerance of 15 ppm and a precursor mass tolerance of 200 Da. The MS/MS and proteoform-spectrum matches were visualized with VisioProt-MS. All the figures were adapted from VisioProt-MS exports using Adobe Illustrator CS6 v16.0.0.

## MS/MS Analysis of Immunopurified STD20s

We analyzed the endogenous 20S immunopurified from human Hek293T-EBNA cells^22^ using intact protein and top-down MS. VisioProt-MS allowed the visualization of the most intense proteoforms based on their deconvoluted MWs (Figure 1A), and most of these were confirmed by MS/MS (Figure 1B). Besides the MS trace, the species selected for MS/MS during the top-down analysis are indicated by empty or filled circles for non-matched MS/MS and proteoform to spectrum matches, respectively. These can be dynamically explored with the “Show data labels” option in the sidebar menu, which triggers dynamic labeling of the data points on hovering. Then, passing the mouse over them informs on the proteoform identified with each MS/MS and their RT, intensity, and deconvoluted MW. It is also possible to highlight the MS/MS matched to specific proteoform(s) of interest such as β6 (PSMB1; Figure 1B, red points). The most intense proteoforms identified in this sample are presented in Table 1 (indicated with the superscript “a”).

Beyond allowing an easy exploration of the identified proteoforms, this representation allows the quick detection of false identifications. For example, in the box “b” (Figure 1B), some MS/MS have been matched to proteoforms in an empty area of the LC-MS map and are most probably background noise. In addition, the single red point overlaid to the β7 subunit MS trace corresponds to an MS/MS that was wrongly matched to β6 (Figure 1B). This could be due to an incorrect estimation of the precursor MW during deconvolution (potential wrong charge state assignment), or a wrong proteoform to spectral matching, and should not be taken into account for further analysis.

In addition to facilitating data curation, the MS/MS mode of VisioProt-MS can be used to optimize the acquisition and search parameters. The dashed rectangle “a” in Figure 1B highlights a proteoform that was not identified by MS/MS. We can see in this figure that several MS/MS were triggered on its elution, but none of them was matched to a protein sequence. This can be due to miss-adapted acquisition methods or search parameters that can be tuned to increase the number of proteoforms identified. In this sample, α7 is mostly phosphorylated, as reported in Gersch et al.^8^

## Comparison of the std20S and i20S

To demonstrate the advantages of VisioProt-MS in the context of comparative sample analysis, we chose the commercial samples of std20S and i20S. Their LC-MS maps are presented in Figure 2, first alone with color-coded intensities (Figure 2A and B) and together for their direct comparison (Figure 2C). These graphical representations indicate the unexpected presence of the subunits β1, β2, and β5 among the i20S-specific subunits of the i20S sample, thereby highlighting a contamination by the std20S (Figure 2B).

Similar graphical representations can be used in other contexts beyond heterogeneous complex analysis, such as identification of covalent drug binding,^8^ monitoring of protein maturation/processing, or identification of context-dependent post-translational modifications or protein variants.^23-25^ Besides zooming and dynamic labeling of the data points, VisioProt-MS allows the user to hide experiment-dependent background noise with the “Threshold” option of the sidebar menu. This defines the percentage of high-intensity MS signal that is visualized on the LC-MS map. It is also possible to mask/unmask sample traces by clicking on their legends, which facilitates the exploration of overlapping signals such as the ones of β5 in Figure 2C.

## Conclusions

For many years, the composition of the 20S core particle of the human proteasome was analyzed with methods such as low-resolution two-dimensional (2D) sodium dodecyl sulfate polyacrylamide gel electrophoresis (SDS-PAGE) separation coupled with analytical techniques (Western blotting or protein/peptide extraction followed by MS analysis).^19,26^ These methods prove the efficiency of 2D representations for mapping proteoforms of complex samples. However, such strategies are experimentally heavy, require a high amount of starting material, and rely on a priori knowledge on the different proteoforms present in the samples. Intact protein and top-down MS remain limited in sensitivity and dynamic range. Furthermore, high-MW proteoforms are still very challenging to study with these techniques. Nevertheless, their ability to precisely measure proteoform MWs and fragment them in a system-wide setup already allows their application to the hypothesis-free exploration of complex samples.^27^ We believe that this could open the doors to personalized medicine at the proteoform level but would rely on technological progresses that go along with an increasing need for dedicated tools to facilitate data analysis.

VisioProt-MS is an easy solution to visualize and inspect intact protein and top-down MS data. It quickly provides an overview of all the detected MWs, reflecting data quality and reproducibility regarding observed MWs, intensities, and RTs. It allows comparison of not only multiple LC-MS runs (including from different deconvolution suites), but also LC-MS and LC-MS/MS runs of the same sample. Furthermore, its dynamic features enable to pinpoint potential new proteoforms, quickly reject wrongly assigned Proteoform Spectral Matches, and spot intense MS signals that remain unassigned.

Today, VisioProt-MS v2.0 is compatible with the following bioinformatics tools: RoWinPro,^8^ Intact Protein Analysis (BioPharma Finder 3.0, Thermo Fisher Scientific), DataAnalysis 4.2 (Bruker),^9^ TopFD (TopPIC Suite),^10^ and ProMex (Informed-Proteomics)^11^ for deconvoluted LC-MS data; and ProSight PD (Proteome Discoverer, Thermo), TopPIC (TopPIC Suite),^10^ and MSPathFinder (Informed-Proteomics)^11^ for the LC-MS/MS data. It is open source and has been developed to be easily adaptable to other formats that may materialize from future technological development. Alternatively, it can be adapted and included in more complex workflows. All the information concerning its features, compatibility, and usages can be found in Locard-Paulet et al^17^ and on the associated online help (https://masstools.ipbs.fr/visioprothelp.html).
